# Standalone Anterior Lumbar Interbody Fusion L5-S1 for Single-Level Degenerative Disc Disease: Implant Parameters Influencing Radiological Results

**DOI:** 10.7759/cureus.101500

**Published:** 2026-01-14

**Authors:** Pierre Barthes, André Boché, Richard Lacroix, Cécile Swennen, Mathieu Severyns, Clément Giraud, Tanguy Vendeuvre

**Affiliations:** 1 Orthopaedics and Traumatology, CHU de Poitiers, Poitiers, FRA; 2 Orthopaedics and Traumatology, University Hospital of Martinique, Fort-de-France, FRA; 3 Statistics, Labcom I3M, University of Poitiers, Poitiers, FRA; 4 Statistics, DACTIM-MIS Team, Laboratoire de Mathématiques Appliquées LMA, CNRS UMR 7348, Poitiers, FRA

**Keywords:** anterior lumbar inter-body fusion, degenerative disc disease, implant parameters, l5-s1, lumbar lordosis, sagittal balance

## Abstract

Introduction

Degenerative disc disease at the L5-S1 level is a common condition and is often associated with chronic low back pain and, in some cases, radicular symptoms. Anterior lumbar interbody fusion (ALIF) is intended to restore segmental and global lumbar lordosis (LL), improve sagittal alignment, and achieve decompression. The primary objective of this study was to examine the association between implant-related parameters and postoperative radiological outcomes following standalone L5-S1 ALIF. A secondary exploratory objective was to assess how closely postoperative LL at the L4-S1 and L5-S1 levels matched pelvic incidence-based theoretical alignment targets. These targets were used as a reference framework for sagittal alignment assessment and were calculated using established proportional relationships between pelvic incidence and LL.

Methods

We conducted a retrospective, single-center observational study based on blinded radiological analysis. Sagittal alignment parameters were assessed using EOS imaging, while interbody fusion was evaluated on one-year postoperative CT scans. A total of 69 adult patients underwent standalone L5-S1 ALIF between January 1, 2017, and January 1, 2023, for single-level degenerative disc disease, without prior spinal instrumentation or deformity. Patients were identified using CCAM (Classification Commune des Actes Médicaux) coding, and eligibility was confirmed through individual chart and imaging review.

Results

Significant postoperative improvements were observed in L1-S1 lordosis (+4.67°), L4-S1 lordosis (+7.1°), L5-S1 lordosis (+9.0°), foraminal height (+3.25 mm), and lumbar distribution index (LDI) (+8.0%) (all p < 0.05). Postoperative L4-S1 lordosis was close to the theoretical target derived from pelvic incidence (mean difference: -0.7° ± 6.6°, p = 0.738), while overcorrection was noted at L5-S1 and undercorrection at L1-S1 (both p < 0.05). Anterior implant height was significantly associated with L4-S1 correction (p = 0.034), as well as with postoperative LDI and foraminal height. One-year CT follow-up showed an 88% fusion rate.

Conclusions

Standalone L5-S1 ALIF was associated with improvements in sagittal alignment by increasing both global and segmental lordosis and by facilitating indirect foraminal decompression. Anterior implant height was the implant parameter most consistently associated with postoperative radiological outcomes, followed by posterior placement and implant depth, while implant lordosis itself was not significantly associated with postoperative alignment.

## Introduction

Degenerative lumbar disc disease (DDD) is a common cause of chronic low back pain and may also result in radicular symptoms. Its prevalence is particularly high at the L5-S1 level, affecting 66.7% of men and 70.9% of women, second only to L4-L5 [1]. When conservative treatment fails, interbody fusion represents an established surgical option [2]. Single-level DDD is often associated with disc height loss and reduced segmental lordosis (SL), which disrupts the natural elliptical distribution of lumbar lordosis (LL). This imbalance has been associated with compensatory hyperlordosis at adjacent levels and increased mechanical stress and may contribute to accelerated degeneration. It is frequently accompanied by reduced foraminal height (FH) and radicular pain [3].

The goals of interbody fusion are to restore both segmental and global lordosis, re-establish sagittal balance, and provide indirect foraminal decompression [4,5]. At the L5-S1 level, the anterior retroperitoneal approach offers specific advantages, including resection of the anterior longitudinal ligament and placement of wedge-shaped implants that enhance segmental correction, provide primary stability, and maximize graft surface area, all contributing to favorable fusion outcomes [6-8].

L5-S1 is a key level in sagittal alignment, contributing approximately 40% of total LL [9]. Correcting distal lordosis at this level is therefore critical to achieving a harmonious distribution of global LL. Moreover, the anterior approach is technically facilitated at this level by favorable vascular anatomy, as the aorto-iliac bifurcation is usually located more cranially. Radiological changes were evaluated after standalone L5-S1 anterior lumbar interbody fusion (ALIF), focusing on L4-S1 lordosis, L5-S1 SL, global lordosis defined as L1-S1 LL, lordosis distribution index (LDI), and FH.

The primary objective of this study was to examine the association between implant-related parameters and postoperative radiological outcomes following standalone L5-S1 ALIF. A secondary exploratory objective was to assess how closely postoperative lordosis at the L4-S1 and L5-S1 levels matched pelvic incidence (PI)-based theoretical alignment targets, which were used as a reference framework for sagittal alignment assessment and were derived from established proportional relationships between PI and LL.

## Materials and methods

Study design

We conducted a monocentric retrospective observational radiological study. We included all patients operated on for single-level ALIF in the L5-S1 spine for DDD between January 1, 2017, and January 1, 2023.

Patient selection

Inclusion criteria were as follows: age >18 years, surgical indication for DDD, and treatment with single-level ALIF at the L5-S1 level. Exclusion criteria were as follows: L5-S1 hinge transition anomaly, presence of spondylolisthesis, pre-existing spinal instrumentation, double-stage arthrodesis, indication for multilevel surgical treatment, and absence of preoperative, postoperative, or follow-up EOS imaging.

Patients were identified retrospectively during the study period using standardized French CCAM (Classification Commune des Actes Médicaux) coding: LFCA005: anterior spinal osteosynthesis and/or arthrodesis without exploration of the canal contents, via laparotomy or lumbotomy; LFFA010: ​​excision of a spinal disc herniation with osteosynthesis and/or arthrodesis, via laparotomy or lumbotomy; LFPA001: ​​anterior osteotomy or complete discectomy for rigid spinal deformity, with arthrodesis and instrumental correction, involving one to three vertebrae, via laparotomy or lumbotomy. Because these codes are not indication-specific, all patients’ medical records and imaging studies were subsequently reviewed by the investigators to confirm the surgical indication and to exclude cases not meeting the predefined inclusion criteria, including multilevel procedures, deformity surgery, spondylolisthesis, or non-degenerative indications.

Surgical technique

All procedures were performed via a retroperitoneal approach by four senior spine surgeons. The main surgical steps were standardized, including patient positioning, approach, disc preparation, and fluoroscopic control. Under general anesthesia and after placement of an indwelling urinary catheter, patients were positioned supine in the Da Vinci (French) position, with the surgeon standing between the patient’s legs. Fluoroscopic localization of the L5-S1 level was first performed in the lateral view, and the surface projection of the L5-S1 disc space was marked on the skin. An anteroposterior fluoroscopic view was then obtained to confirm correct patient positioning and ensure pedicle symmetry. A vertical skin incision was made centered over the projected L5-S1 disc space.

Careful dissection was carried out to identify the linea alba. A right-sided paramedian incision was performed lateral to the linea alba to expose the rectus abdominis muscle and allow a right-sided retroperitoneal approach. Blunt and meticulous dissection was continued to identify the vascular plane, with mobilization of the vessels on either side of the L5-S1 disc space. Lateral fluoroscopy was used to confirm the correct operative level. After resection of the anterior longitudinal ligament, a complete discectomy was performed. The posterior longitudinal ligament was ruptured using an interbody distractor and a dilator, and subsequently resected using a Kerrison rongeur. The vertebral endplates were carefully prepared using curettes. Implant selection was not fully standardized and was based on intraoperative stability and press-fit assessment, according to surgeon preference and anatomical constraints. Final fluoroscopic controls were then performed to confirm proper implant centering and positioning (maximum endplate coverage).

Data collection

**Figure 1:**
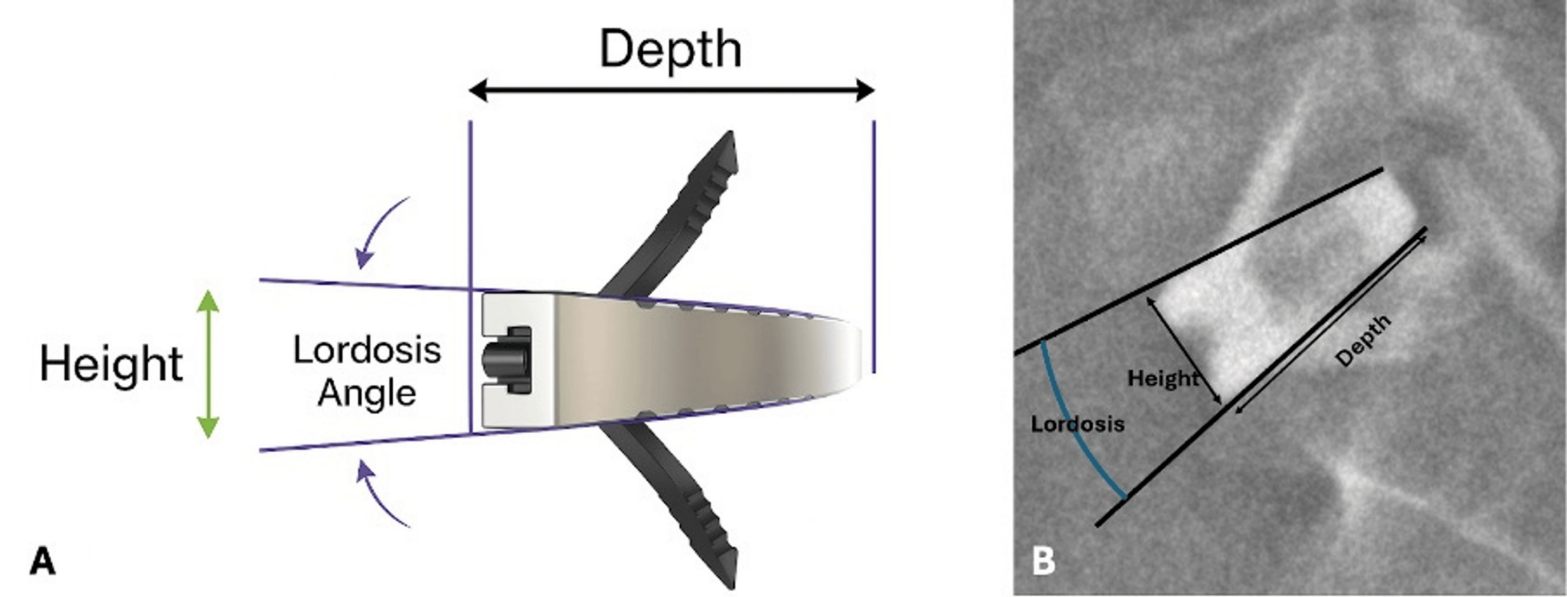
Parameters of anterior lumbar interbody fusion implants Height (mm), depth (mm), and lordosis angle (°) (A). Radiological correlation of these parameters at the L5-S1 level on EOS imaging (B)

Demographic variables (sex, height, weight, BMI, age) and implant parameters (Figure 1) were retrieved from the electronic medical records.

All patients underwent a preoperative as well as a postoperative EOS between two months and one year after surgery. EOS imaging was performed using a standardized biplanar low-dose acquisition system, providing simultaneous anteroposterior and lateral full-spine radiographs. All examinations were acquired with the patient in a relaxed standing position, barefoot, with knees fully extended and hands resting on the shoulders, in accordance with standard EOS acquisition protocols. On the anteroposterior and lateral views, the femoral heads were identified bilaterally to determine the bicoxofemoral axis, which served as the reference for pelvic measurements. The sacrum was visualized in its entirety, enabling accurate identification of the sacral endplate. Pelvic parameters were measured using established anatomical landmarks, including the center of the femoral heads, the midpoint of the sacral endplate, and the vertical reference line.

PI, pelvic tilt (PT), and sacral slope (SS) were calculated according to conventional definitions. Lumbar lordosis measurements were performed on the lateral view using the Cobb method: global lordosis was defined as the angle between the superior endplate of L1 and the superior endplate of S1 (L1-S1), distal lordosis as the angle between L4 and S1 (L4-S1), and segmental lordosis as the angle between the inferior endplate of L5 and the superior endplate of S1 (L5-S1). Sagittal vertical axis (SVA) was measured as the horizontal distance between the C7 plumb line and the posterior superior corner of S1. All measurements were performed using sterEOS software (EOS Imaging), which allows three-dimensional reconstruction and precise angular and linear assessment from biplanar radiographs.

**Figure 2:**
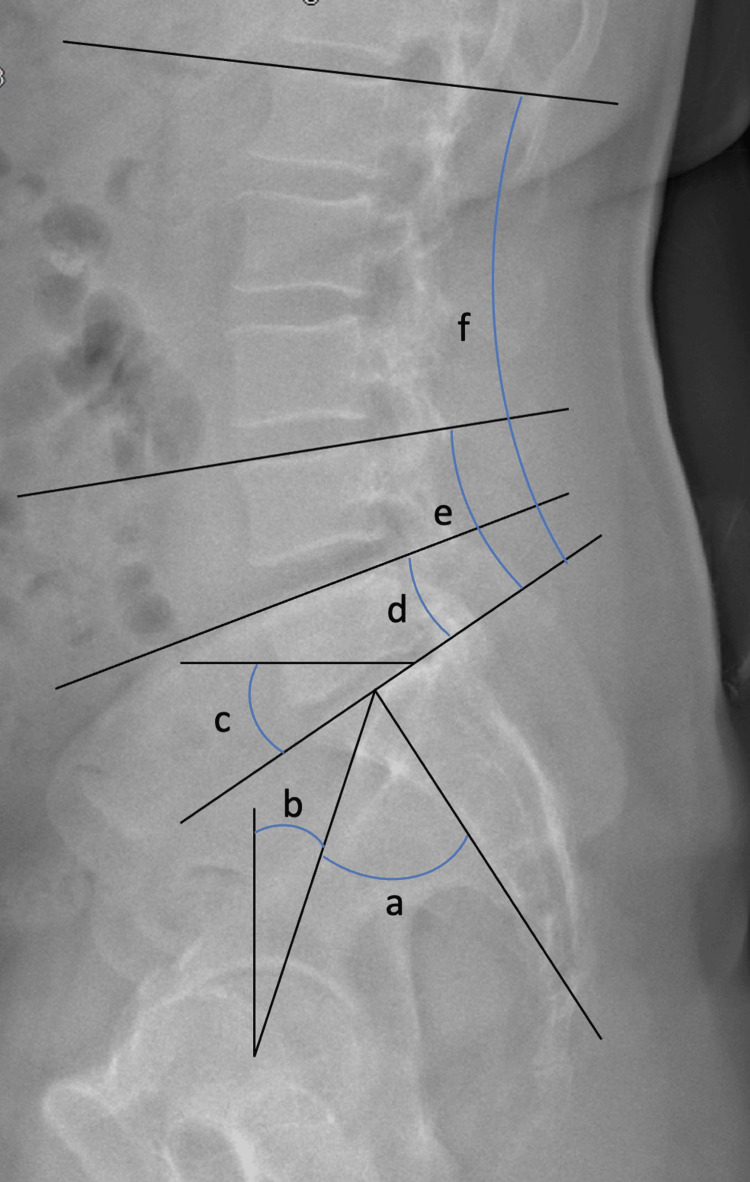
Pelvic parameters and lumbar lordosis measurements on EOS lateral view a: pelvic incidence (PI, °); b: pelvic tilt (PT, °); c: sacral slope (SS, °); d: L5-S1 lordosis (°); e: L4-S1 lordosis (°); f: L1-S1 lordosis (°)

**Figure 3:**
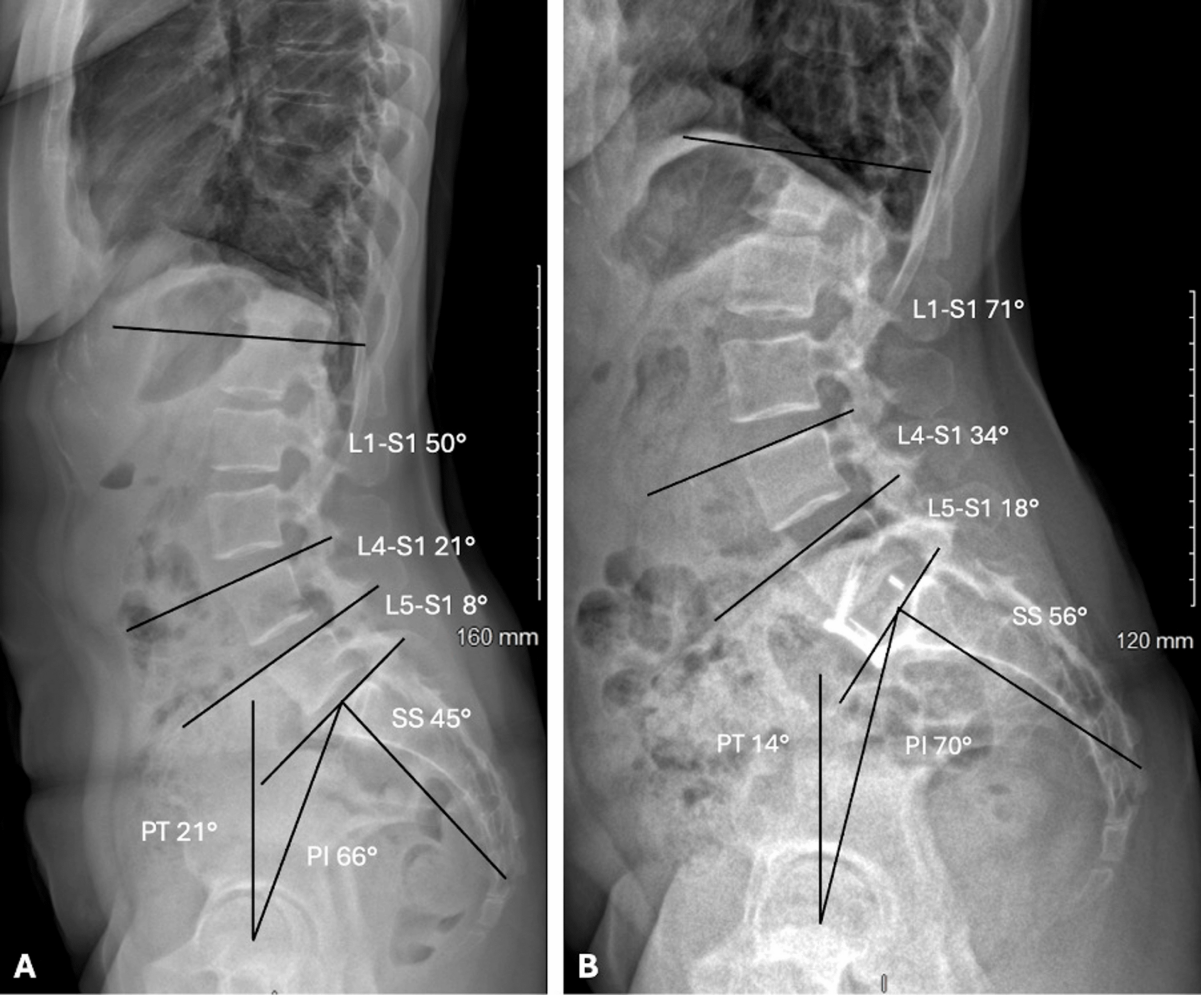
Preoperative (A) and postoperative EOS measurements (B) Pelvic incidence (PI, °); pelvic tilt (PT, °); sacral slope (SS, °); L5-S1 lordosis (°); L4-S1 lordosis (°); L1-S1 lordosis (°)

Radiological measurements (Figures 2, 3) were carried out by a single blinded independent observer using sterEOS software: L1-S1, L4-S1, and L5-S1 lordosis (°), SVA (mm), PI (°), SS (°), PT (°), anterior disc height (ADH, mm), posterior disc height (PDH, mm), FH (mm), L5 lower endplate depth (mm), S1 upper endplate depth (mm), and distance between the anterior L5 endplate and the implant (mm). Interbody fusion was assessed on one-year postoperative CT imaging using the Bridwell fusion grading system (grade I-fused with remodelling and trabeculae present; grade II-graft intact, not fully remodelled and incorporated, but no lucency present; grade III-graft intact, potential lucency present at top and bottom of graft; grade IV-fusion absent with collapse/resorption of the graft) [10]. No inter- or intra-observer reliability assessment was performed.

Theoretical target lordosis (TTL) was determined for each patient based on preoperative PI. We introduced 'coverage rates' (CR), which represent the extent of intervertebral implant coverage in the anteroposterior plane. These CR values will be tested as positioning parameters in the correlation analyses. The following formulas were used: theoretical L1-S1 lordosis (°) = 0.54 x PI (°) + 32.56 [11]; theoretical L4-S1 lordosis (°) = 0.66 x L1-S1 lordosis (°) [12]; theoretical L5-S1 lordosis (°) = 0.4 x L1-S1 lordosis (°) [9]; LDI (%) = L4-S1 lordosis (°)/L1-S1 lordosis (°) [13,14]; CR = Implant depth (in mm)/L5 endplate depth (mm); corrected CR = (implant depth (mm) + cage distance from L5 anterior wall (mm))/L5 endplate depth (mm)

Statistical analysis

Statistical analysis was performed using R software. Data were expressed as means ± standard deviations (SD), or as numbers (percentages). Non-parametric statistical methods were used because the data did not follow a normal distribution. Therefore, t-tests, ANOVA, and chi-square tests were not applicable. Paired Wilcoxon tests were used for statistical analysis of preoperative and postoperative results and for comparisons with theoretical targets, to enhance test sensitivity. Correlation analyses were performed using Spearman's rank correlation coefficients. Comparisons of results in PI subgroups were performed using Kruskal-Wallis and Fisher’s exact tests. P-values < 0.05 were considered significant. Analyses were performed on a complete-case basis. Comparisons with pelvic incidence-based theoretical lordosis targets were performed for descriptive and exploratory purposes only, as these targets are model-derived reference values rather than observed paired measurements.

## Results

**Figure 4:**
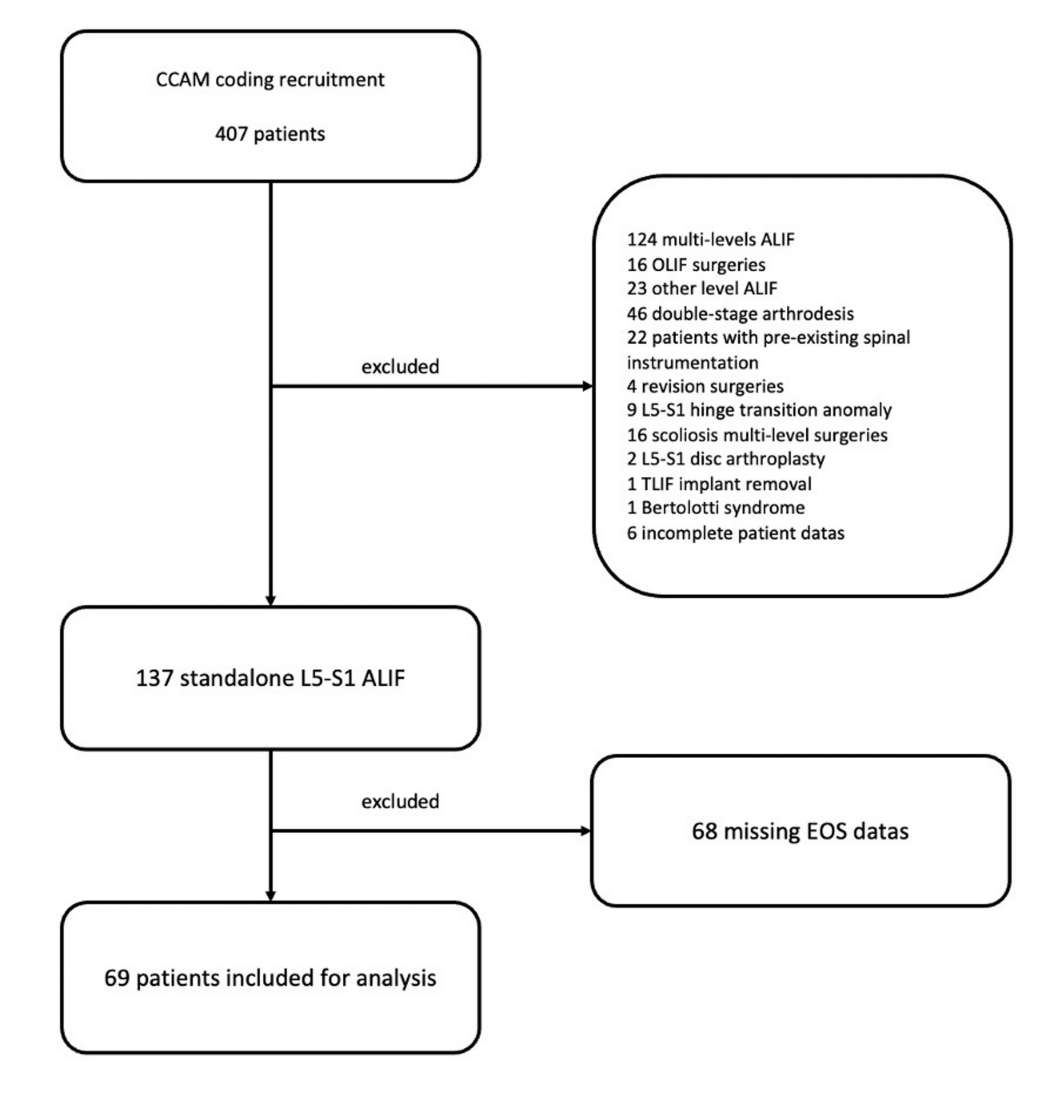
Flow chart depicting patient selection from initial recruitment to final analysis CCAM: Classification Commune des Actes Médicaux (French coding); OLIF: oblique lateral interbody fusion; ALIF: anterior lumbar interbody fusion; TLIF: transforaminal lumbar interbody fusion

All in all, 407 patients were screened, of whom 137 underwent a single-level L5-S1 ALIF. Because of missing EOS data in 68 patients, a total of 69 patients were ultimately included in the analysis (Figure 4).

The mean age of the cohort was 46.7 years (range: 29-75 years), with a sex ratio of 46F/23M, and a mean BMI of 27.9 kg/m2 (range: 18.8-37.8 kg/m2). The implant anterior height averaged 14.42 mm (range: 12-18 mm), implant lordosis averaged 14.55° (range: 10-20°), and implant depth averaged 28.38 mm (range: 26-34 mm) (Table 1).

**Table 1:** Patients and material - baseline characteristics BMI: body mass index; SD: standard deviation

Baseline characteristics	Values
Age (years) mean ± SD	46.7 ± 9.1
Sex ratio	46 F/23 M
Height (cm) mean ± SD	169.3 ± 8.6
Weight (kg) mean ± SD	80.2 ± 15.1
BMI (kg/m²) mean ± SD	27.9 ± 4.6
Implants (n = 69)	
Zero-profile titanium implants with screws fixation, n	16
Cage + plate peek implants, n	53
Implant anterior height (mm) mean ± SD	14.42 ± 1.4
Implant lordosis (°) mean ± SD	14.55 ± 2.2
Implant depth (mm) mean ± SD	28.38 ± 2.0

Radiological results

**Figure 5:**
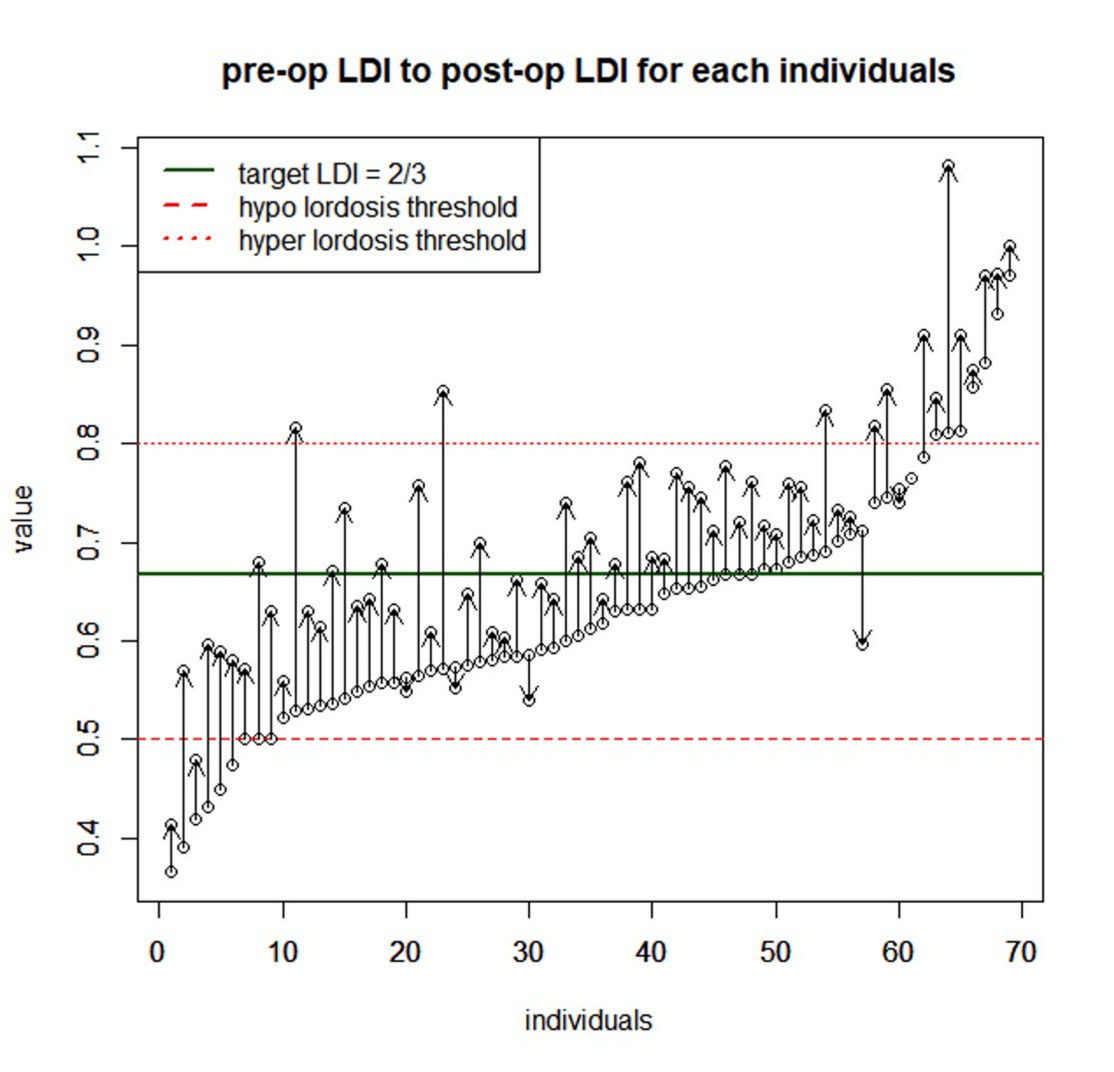
Changes in LDI for each patient between pre- and post-op LDI <50% corresponds to malalignment with hypo-lordosis of the caudal segment. LDI between 50% and 80% is considered satisfactory alignment. LDI >80% corresponds to malalignment with hyperlordosis of the caudal segment LDI: lordosis distribution index

We found a significant (p < 0.05) increase in PI of 3.51° ± 3.86°, SS of 4.06° ± 5.00°, ADH of 8.71 mm ± 3.33 mm, PDH of 4.15 mm ± 2.06 mm, FH of 3.25 mm ± 2.07 mm, L1-S1 lordosis of 4.67° ± 6.47°, L4-S1 lordosis of 7.10° ± 5.97°, L5-S1 lordosis of 9.01° ± 5.58°, and LDI of 8% ± 6.9% (Table 2, Figure 5).

**Table 2:** Preoperative and postoperative radiological results Paired Wilcoxon tests (p-value) were performed SD: standard deviation; SVA: sagittal vertical axis; ADH: anterior disc height; PDH: posterior disc height; LDI: lordosis distribution index

	Preoperative measurements	Postoperative measurements	P-value
Pelvic Incidence (°), mean ± SD	47.46 ± 9.71	50.97 ± 10.81	<0.05
Pelvic tilt (°), mean ± SD	11.72 ± 6.18	11.06 ± 6.81	0.082
Sacral slope (°), mean ± SD	35.72 ± 7.91	39.78 ± 8.59	<0.05
SVA (mm), mean ± SD	23.96 ± 28.06	20.61 ± 26.91	0.215
ADH (mm), mean ± SD	7.1 ± 2.74	15.81 ± 1.99	<0.05
PDH (mm), mean ± SD	2.61 ± 1.32	6.76 ± 1.93	<0.05
Foraminal height (mm), mean ± SD	13.8 ± 2.1	17.04 ± 2.51	<0.05
L1-S1 lordosis (°), mean ± SD	50.2 ± 10.22	54.87 ± 11.11	<0.05
L4-S1 lordosis (°), mean ± SD	31.01 ± 6.71	38.12 ± 6.29	<0.05
L5-S1 lordosis (°), mean ± SD	16.93 ± 5.15	25.94 ± 5.2	<0.05
L5-S1/L1-S1, mean ± SD	0.34 ± 0.12	0.49 ± 0.11	<0.05
LDI (%), mean ± SD	63 ± 12	71 ± 12	<0.05
Subgroup LDI (n = 69)			
<50%	6	2	
50-80%	56	54	
>80%	7	13	

**Figure 6:**
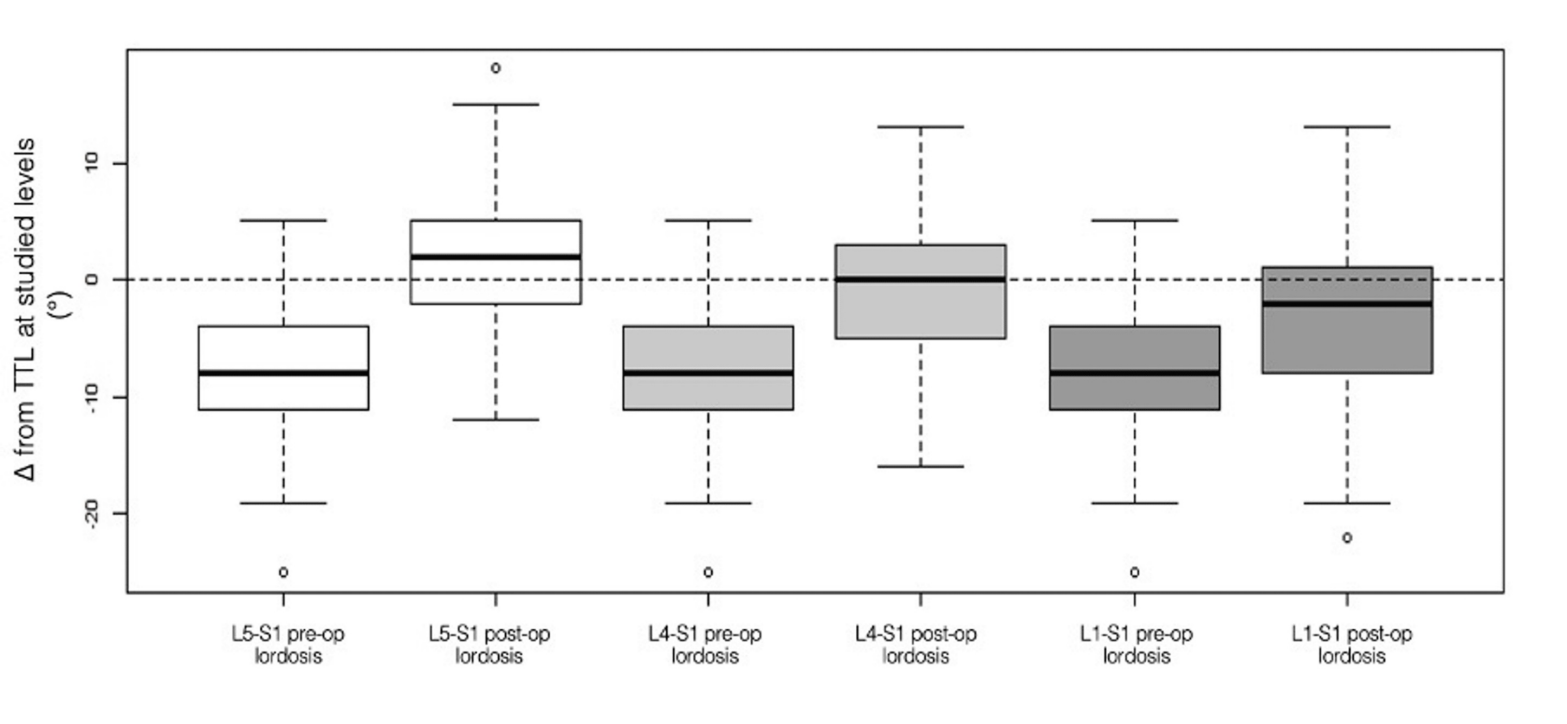
Preoperative and postoperative deviations from TTL at L5-S1, L4-S1, and L1-S1 This figure displays box plots illustrating the difference (Δ) between measured lordosis and the TTL at the L5-S1, L4-S1, and L1-S1 levels, both preoperatively and postoperatively. Negative values reflect undercorrection relative to TTL, whereas positive values reflect overcorrection. Δ was calculated as measured lordosis minus TTL, with TTL derived from pelvic incidence-based formulas. The figure highlights the magnitude of correction achieved at each level and shows a progressive decrease in correction efficiency at more proximal lumbar segments TTL: theoretical target lordosis

The main hypothesis was tested using paired Wilcoxon tests and value distribution: postoperative L5-S1 lordosis was greater than TTL (p = 0.00027), postoperative L4-S1 lordosis was not statistically different from TTL (p = 0.7378), and postoperative L1-S1 lordosis was lower than TTL (p = 0.0176) (Table 3, Figure 6).

**Table 3:** Differences between measured lordosis and TTL at L1-S1, L4-S1, and L5-S1 Levels TTL was calculated from pelvic incidence. Positive Δ values indicate lordosis greater than the theoretical target, and negative Δ values indicate lordosis lower than the theoretical target TTL: theoretical target lordosis; SD: standard deviation

Variables	Values, mean ± SD
Theoretical L1-S1 lordosis (°)	58.2° ± 5.2
Δ L1-S1 preoperative – TTL (°)	-8.0° ± 8.7
Δ L1-S1 postoperative – TTL (°)	-3.32° ± 9.5
Theoretical L4-S1 lordosis (°)	38.8° ± 3.5
Δ L4-S1 preoperative – TTL (°)	-7.8° ± 7.0
Δ L4-S1 postoperative – TTL (°)	-0.7° ± 6.5
Theoretical L5-S1 lordosis (°)	23.3° ± 2.1
Δ L5-S1 preoperative – TTL (°)	-6.35° ± 5.7
Δ L5-S1 postoperative – TTL (°)	2.67° ± 5.6

Interbody fusion was evaluated on postoperative CT scans using the modified Bridwell fusion grading system [10]. Among the 69 patients, 37 (54%) achieved Grade I fusion, and 24 (35%) achieved Grade II fusion, resulting in an overall satisfactory fusion rate of 88%. Grade III fusion was observed in seven patients (10%), and one patient (1%) demonstrated Grade IV nonunion.

Correlation analyses

**Figure 7:**
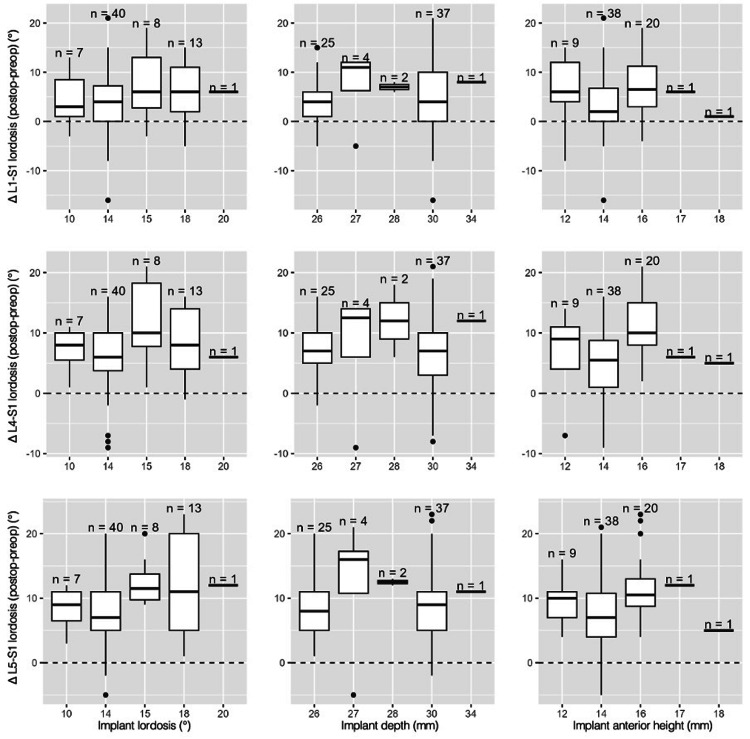
Box plots illustrating the distribution of implant parameters (anterior height, depth, and implant lordosis) in relation to postoperative–preoperative lordosis differentials at each studied spinal level (L5-S1, L4-S1, L1-S1) Boxplots illustrating the relationship between implant parameters and postoperative changes in lordosis: Δ L5-S1 lordosis (top row), Δ L4-S1 lordosis (middle row), and Δ L1-S1 lordosis (bottom row). Columns represent implant lordosis (°), implant depth (mm), and anterior implant height (mm). Sample sizes for each subgroup are indicated above the boxplots. The dashed horizontal line represents no change (Δ = 0°)

We looked for a correlation between implant parameters (IAH, implant lordosis, and implant depth) and positioning parameters (CR, corrected CR, and the distance from the L5 anterior wall) with postoperative results (L1-S1, L4-S1, and L5-S1 lordosis, LDI, and FH), as well as their postoperative to preoperative and postoperative to theoretical lordosis differentials (Figure 7).

The associations found were as follows: IAH and Δ postoperative-preoperative L4-S1 lordosis (ρ = 0.256, p = 0.034); IAH and Δ postoperative-preoperative LDI (ρ = 0.362, p = 0.002); IAH and postoperative FH (ρ = 0.309, p = 0.0099); corrected CR and Δ postoperative-preoperative FH (ρ = 0.291, p = 0.015)

**Figure 8:**
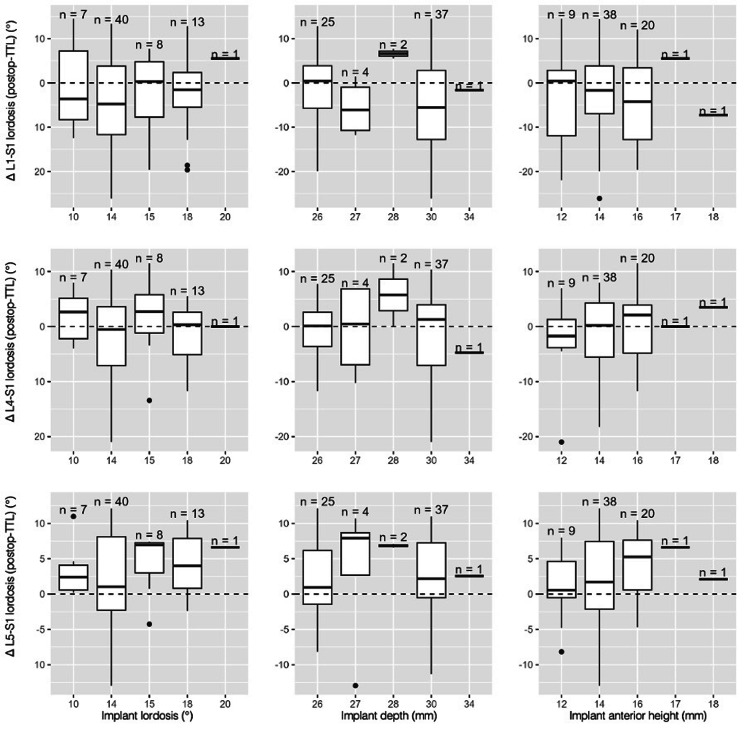
Box-plots illustrating the distribution of implant parameters (anterior height, depth, and cage lordosis) in relation to postoperative-theoretical lordosis differentials at each studied spinal level (L5-S1, L4-S1, L1-S1) Boxplots illustrating postoperative deviation from the theoretical target lordosis (Δ postoperative – TTL) at three levels: L5-S1 (top row), L4-S1 (middle row), and L1-S1 (bottom row). Columns correspond to implant characteristics: implant lordosis (°), implant depth (mm), and implant anterior height (mm). Sample sizes for each implant subgroup are displayed above the boxplots. The horizontal dashed line represents perfect alignment with the theoretical target (Δ = 0°). Values above the line indicate overcorrection, while values below indicate undercorrection TTL: theoretical target lordosis

In our population, greater implant anterior height (IAH) was associated with a larger postoperative-preoperative difference in L4-S1 lordosis, LDI, and postoperative FH. Additionally, greater cage depth and more posterior implant positioning were linked to larger postoperative-preoperative differences in FH. No other correlation tests showed statistically significant results (Figure 8).

Analysis of radiological results by PI subgroups

The mean preoperative PI was 47.46° ± 9.71° (Table 2). The three subgroups of preoperative PI (PI < 45°: 24 patients, PI 45-60°: 37 patients, and PI > 60°: eight patients) were comparable in terms of patient and implant characteristics (Table 4). We found significant differences between the subgroups for L1-S1 lordosis, PI, PT, SS, and LDI both in preoperative and postoperative measurements (Table 5).

**Table 4:** Patient characteristics, implant parameters, and positioning variables across pelvic incidence subgroups Comparisons were performed using the Kruskal-Wallis test (H-statistic and p-value) and Fisher’s exact test (p-value) SD: standard deviation; NA: not applicable

Pelvic incidence subgroups	<45^°^	45-60^°^	>60^°^	Overall	P-value	H-statistic
Patient characteristics						
Sex F, n (%)	16 (66.67)	23 (62.16)	7 (87.5)	46 (66.67)	0.44278	NA
Sex M, n (%)	8 (33.33)	14 (37.84)	1 (12.5)	23 (33.33)	0.44278	NA
Weight (kg), mean ± SD	81.62 ± 17.91	80.11 ± 14.17	76.25 ± 11.61	80.19 ± 15.2	0.74824	0.5800693
Height (cm), mean ± SD	169.5 ± 9.33	169.38 ± 8.86	168.88 ± 6.15	169.36 ± 8.66	0.98921	0.0216985
BMI (kg/m^2^), mean ± SD	28.31 ± 5.42	27.91 ± 4.39	26.64 ± 3.26	27.9 ± 4.64	0.76067	0.5471058
Age at surgery (years), mean ± SD	45.04 ± 7.94	48.57 ± 9.48	43.25 ± 10.5	46.72 ± 9.19	0.21059	3.1156527
Implant parameters						
Cage + plate peek implants, n (%)	20 (83.3)	26 (70.27)	7 (87.5)	53 (76.81)	0.27536	NA
Zero-profile titanium implants, n (%)	4 (16.7)	11 (29.73)	1 (12.5)	16 (23.19)	0.27536	NA
Implant depth (mm), mean ± SD	28.92 ± 2.1	28.11 ± 1.93	28 ± 2.14	28.38 ± 2.02	0.28288	2.5254899
Implant anterior height (mm), mean ± SD	14.17 ± 1.43	14.62 ± 1.36	14.25 ± 1.28	14.42 ± 1.38	0.49645	1.4005443
Implant lordosis (°), mean ± SD	14.21 ± 2.5	14.54 ± 2.1	15.62 ± 2	14.55 ± 2.25	0.27707	2.5669918
Vertebra parameters						
Inferior L5 endplate depth (mm), mean ± SD	33.42 ± 2.36	33.19 ± 3.52	32 ± 3.07	33.13 ± 3.1	0.4067	1.7993819
Superior S1 endplate depth (mm), mean ± SD	33.12 ± 3.66	33.59 ± 3.41	31.75 ± 2.43	33.22 ± 3.41	0.41176	1.7746207
L5 S1 discal lordosis pre-op (°), mean ± SD	6.12 ± 4.48	7.38 ± 5.41	6.38 ± 4.96	6.83 ± 5.02	0.41234	1.7718182
Implant positioning						
Implant anterior wall to anterior L5 wall (mm), mean ± SD	-4.25 ± 1.78	-3.05 ± 3.23	-4.38 ± 1.77	-3.62 ± 2.7	0.2709	2.6120014
Coverage ratio, mean ± SD	0.88 ± 0.08	0.86 ± 0.07	0.88 ± 0.1	0.87 ± 0.08	0.37986	1.9359310
Corrected coverage ratio, mean ± SD	0.75 ± 0.08	0.76 ± 0.11	0.74 ± 0.1	0.76 ± 0.1	0.93831	0.1273519

**Table 5:** Pelvic incidence subgroup analyses for preoperative, postoperative, and theoretical target measurements Kruskal-Wallis tests include both p-values and H-statistic; Fisher’s exact tests include p-values only SD: standard deviation; SVA: sagittal vertical axis; PI: pelvic incidence; NA: not applicable; PT: pelvic tilt; SS: sacral slope; ADH: anterior disc height; PDH: posterior disc height; FH: foraminal height; LDI: lordosis distribution index; TTL: theoretical target lordosis

Pelvic incidence subgroups	<45^°^	45-60^°^	>60^°^	Overall	P-value	H-statistic
Preoperative measurements						
L1-S1 lordosis (°), mean ± SD	44.54 ± 8.26	51.57 ± 9.63	60.88 ± 7.95	50.2 ± 10.22	0.00022	16.832056
L5-S1 lordosis (°), mean ± SD	17.29 ± 4.09	16.7 ± 5.71	16.88 ± 5.84	16.93 ± 5.15	0.95983	0.0819922
L4-S1 lordosis (°), mean ± SD	29.92 ± 5.55	31.08 ± 7.08	34 ± 8	31.01 ± 6.71	0.29882	2.4158404
SVA (mm), mean ± SD	18.5 ± 24.15	27.78 ± 30.1	22.62 ± 29.86	23.96 ± 28.06	0.30255	2.3910063
PI (°), mean ± SD	37.21 ± 4.39	50.35 ± 4.1	64.88 ± 3.83	47.46 ± 9.71	<0.0001	54.683878
PT (°), mean ± SD	8.17 ± 5.1	12.7 ± 5.45	17.88 ± 6.4	11.72 ± 6.18	0.00043	15.508122
SS (°), mean ± SD	28.96 ± 5.17	37.65 ± 5.54	47.12 ± 6.1	35.72 ± 7.91	<0.0001	35.182577
ADH (mm), mean ± SD	7 ± 2.77	7.08 ± 2.99	7.5 ± 1.31	7.1 ± 2.74	0.96435	0.0726004
PDH (mm), mean ± SD	2.5 ± 1.29	2.54 ± 1.32	3.25 ± 1.39	2.61 ± 1.32	0.31362	2.3191286
FH (mm), mean ± SD	13.71 ± 1.9	13.54 ± 2.18	15.25 ± 1.98	13.8 ± 2.1	0.19204	3.3000754
L5.S1/L1.S1 lordosis ratio, mean ± SD	0.4 ± 0.11	0.32 ± 0.12	0.27 ± 0.08	0.34 ± 0.12	0.00636	10.116546
LDI, mean ± SD	0.68 ± 0.14	0.61 ± 0.1	0.55 ± 0.08	0.63 ± 0.12	0.01175	8.8879704
Postoperative measurements						
L1-S1 lordosis, mean ± SD	49.12 ± 9.22	55.81 ± 10.21	67.75 ± 8.71	54.87 ± 11.11	0.00026	16.515384
L5-S1 lordosis, mean ± SD	26.42 ± 4.37	25.3 ± 5.67	27.5 ± 5.42	25.94 ± 5.2	0.50058	1.3839854
L4-S1 lordosis, mean ± SD	36.96 ± 5.13	38.14 ± 6.32	41.5 ± 8.67	38.12 ± 6.29	0.31752	2.2944017
SVA (mm), mean ± SD	13.88 ± 26.21	22.35 ± 26.63	32.75 ± 28.14	20.61 ± 26.91	0.30348	2.3848846
PI (°), mean ± SD	41.25 ± 5.12	52.65 ± 5.28	72.38 ± 6.61	50.97 ± 10.81	<0.0001	47.826572
PT (°), mean ± SD	7.71 ± 6.56	11.54 ± 5.14	18.88 ± 8.03	11.06 ± 6.81	0.00082	14.210122
SS (°), mean ± SD	33.33 ± 5.9	41.05 ± 6.26	53.25 ± 6.52	39.78 ± 8.59	<0.0001	30.687371
ADH (mm), mean ± SD	15.42 ± 1.82	15.95 ± 1.86	16.38 ± 3.02	15.81 ± 1.99	0.63619	0.9045228
PDH (mm), mean ± SD	6.62 ± 1.86	6.72 ± 2.08	7.38 ± 1.51	6.76 ± 1.93	0.60422	1.0076486
FH (mm), mean ± SD	16.67 ± 2.48	16.92 ± 2.63	18.75 ± 1.16	17.04 ± 2.51	0.07416	5.2031456
L5.S1/L1.S1 lordosis ratio, mean ± SD	0.55 ± 0.1	0.46 ± 0.1	0.41 ± 0.08	0.49 ± 0.11	0.00238	12.077400
LDI, mean ± SD	0.77 ± 0.13	0.69 ± 0.11	0.61 ± 0.09	0.71 ± 0.12	0.00402	11.032845
Postoperative LDI subgroups						
<0.80, n (%)	16 (66.67)	32 (86.49)	8 (100)	56 (81.16)	0.06697	NA
>0.80, n (%)	8 (33.33)	5 (13.51)	0 (0)	13 (18.84)	0.06697	NA
TTL values						
L5-S1 lordosis TTL (°), mean ± SD	21.06 ± 0.95	23.9 ± 0.89	27.04 ± 0.83	23.28 ± 2.1	<0.0001	54.683878
L4-S1 lordosis TTL (°), mean ± SD	35.1 ± 1.58	39.83 ± 1.48	45.06 ± 1.38	38.79 ± 3.5	<0.0001	54.683878
L1-S1 lordosis TTL (°), mean ± SD	52.65 ± 2.37	59.75 ± 2.22	67.59 ± 2.07	58.19 ± 5.24	<0.0001	54.683878
Δ L1-S1 postoperative-TTL, mean ± SD	-3.53 ± 8.85	-3.94 ± 10.07	0.16 ± 9.88	-3.32 ± 9.59	0.53617	1.2465902
Δ L4-S1 postoperative-TTL, mean ± SD	1.86 ± 5.03	-1.7 ± 6.42	-3.56 ± 9.6	-0.68 ± 6.61	0.0492	6.0236705
Δ L5-S1 postoperative-TTL, mean ± SD	5.36 ± 4.48	1.4 ± 5.75	0.46 ± 5.76	2.67 ± 5.63	0.01669	8.1856873

Postoperative-theoretical differentials were statistically significant only for L4-S1 and L5-S1 (Table 5). The only significant postoperative-preoperative difference observed was in PI (Table 6).

**Table 6:** Postoperative-preoperative differentials stratified by PI subgroups Statistical comparisons were performed using the Kruskal-Wallis test; p-values and corresponding H-statistic are reported PI: pelvic incidence; SD: standard deviation; ADH: anterior disc height; PDH: posterior disc height; FH: foraminal height; PS: pelvic slope; PT: pelvic tilt; SVA: sagittal vertical axis; LDI: lordosis distribution index

Pelvic incidence subgroups/postoperative-preoperative Δ	<45^°^, mean ± SD	45-60^°^, mean ± SD	>60^°^, mean ± SD	Overall	P-value	H-statistic
L1-S1 lordosis (°)	4.58 ± 5.32	4.24 ± 6.52	6.88 ± 9.75	4.67 ± 6.52	0.79066	0.4697833
L4-S1 lordosis (°)	7.04 ± 4.14	7.05 ± 6.39	7.5 ± 9.21	7.1 ± 6.02	0.96975	0.0614319
L5-S1 lordosis (°)	9.12 ± 4.15	8.59 ± 6.37	10.62 ± 6.14	9.01 ± 5.62	0.76227	0.5429054
ADH (mm)	8.42 ± 3.03	8.86 ± 3.6	8.88 ± 3.44	8.71 ± 3.35	0.92293	0.1604019
PDH (mm)	4.12 ± 2.21	4.18 ± 2.11	4.12 ± 1.64	4.15 ± 2.07	0.98802	0.0241105
FH (mm)	2.96 ± 2.12	3.38 ± 2.1	3.5 ± 2.07	3.25 ± 2.08	0.58317	1.0785480
PS (°)	4.38 ± 4.08	3.41 ± 4.9	6.12 ± 7.81	4.06 ± 5.04	0.60695	0.9986186
PT (°)	-0.46 ± 3.72	-1.16 ± 3.24	1 ± 5.21	-0.67 ± 3.68	0.28392	2.5181343
PI (°)	4.04 ± 2.65	2.3 ± 3.71	7.5 ± 5.1	3.51 ± 3.89	0.00089	14.044659
SVA (mm)	-4.62 ± 27.92	-5.43 ± 24.35	10.12 ± 24.48	-3.35 ± 25.76	0.32006	2.2784652
L5-S1/L1-S1 ratio	0.15 ± 0.06	0.14 ± 0.12	0.14 ± 0.07	0.14 ± 0.1	0.21485	3.0756673
LDI	0.08 ± 0.07	0.09 ± 0.07	0.06 ± 0.07	0.08 ± 0.07	0.49523	1.4054474

**Figure 9:**
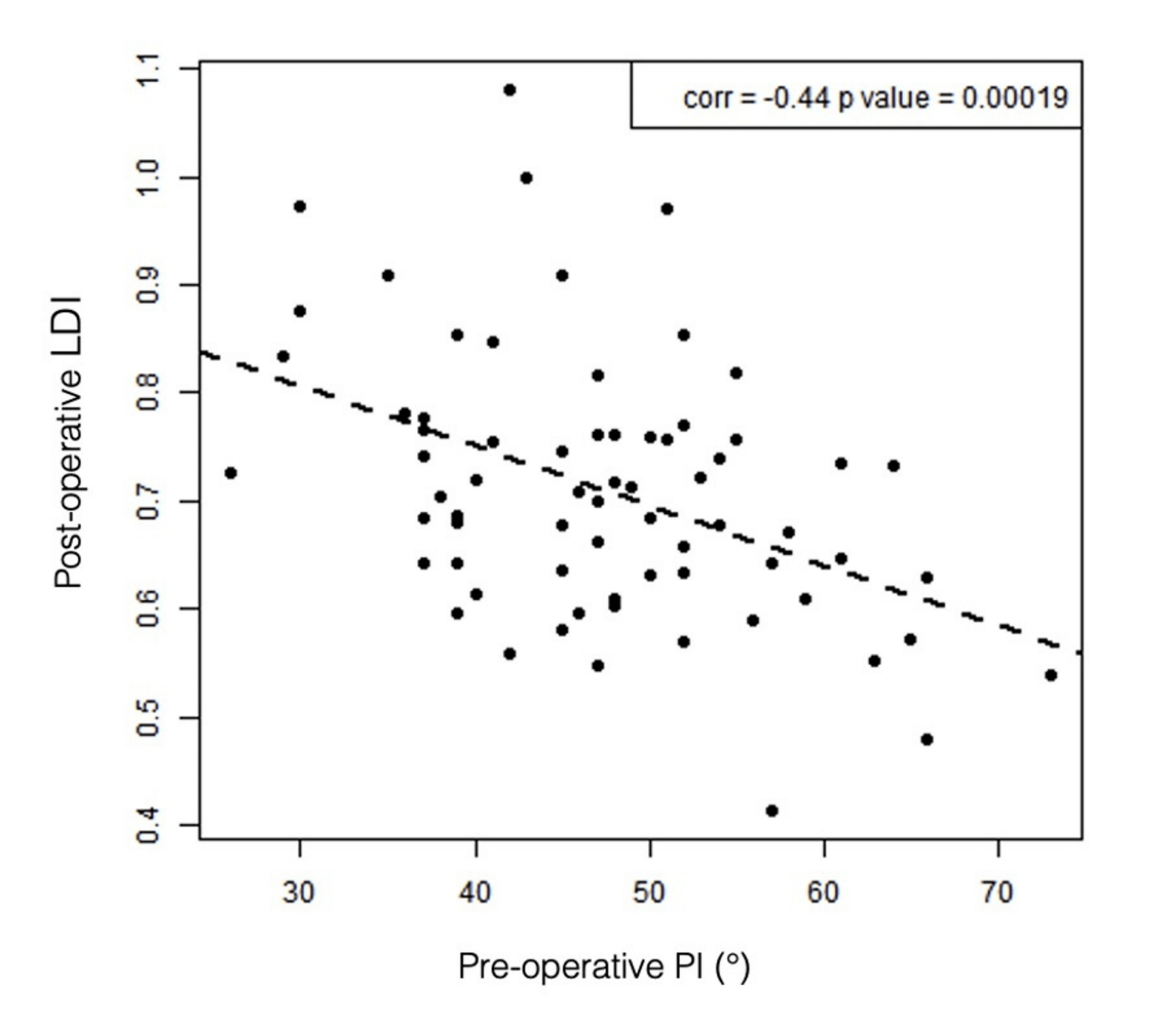
Association between preoperative PI and postoperative LDI Scatterplot illustrating the association between preoperative PI (°) and postoperative LDI, with each point representing an individual patient. The dashed line represents the linear regression fit. The correlation coefficient and corresponding p-value (r = -0.44; p = 0.00019) are displayed in the upper right corner PI: pelvic incidence; LDI: lordosis distribution index

We found that preoperative pelvic incidence was inversely correlated with postoperative LDI (p < 0.001 ρ: -0.435 IC [-0.609; -0.221]). Higher pelvic incidence was associated with lower postoperative LDI values in our population (Figure 9).

## Discussion

The main goals of this study were to explore radiological changes after standalone L5-S1 ALIF and to examine associations between implant parameters and postoperative sagittal alignment. Using a pelvic incidence-based reference framework, we secondarily assessed how postoperative L4-S1 lordosis compared to theoretical alignment targets derived from established proportional relationships between pelvic incidence and lumbar lordosis. These targets were used as a descriptive reference rather than validated surgical endpoints. While similar alignment-focused ALIF studies have been previously reported with different conceptual approaches, the present analysis provides an additional perspective by contextualizing postoperative alignment within a PI-based proportional framework. This series also represents one of the largest cohorts of standalone L5-S1 ALIF procedures for degenerative disc disease with detailed sagittal alignment assessment.

Our findings support the corrective potential of standalone L5-S1 ALIF for sagittal alignment. Significant improvements were observed at multiple levels: L5-S1 lordosis increased by +9.0°, L4-S1 lordosis matched closely with the TTL (+7.1°), and L1-S1 lordosis showed a more moderate gain (+4.7°). Lumbar lordosis distribution improved, with an increase in the LDI, while FH also increased, supporting the efficacy of ALIF in achieving indirect foraminal decompression. However, correction efficiency declined at higher lumbar levels, likely reflecting reduced hyperextension compensation proximally. Although these lordosis gains reached statistical significance, their magnitude should be interpreted cautiously, taking into account the inherent measurement variability of sagittal alignment parameters and the fact that statistically significant changes do not necessarily translate into clinically meaningful differences. These comparisons should be interpreted with caution, as PI-based theoretical targets are derived from population-based proportional models. The present analysis specifically examined postoperative L4-S1 lordosis in the context of theoretical caudal alignment, intending to describe lordosis distribution patterns in patients with degenerative disc disease at the lumbosacral junction. Such restoration of a harmonious lordosis profile may be of interest, as it has been associated with a lower risk of subsequent mechanical complications in previous studies [14-16].

Comparison with previous studies highlights both similarities and differences. Formica et al. reported moderate increases in L1 to S1 and segmental lordosis, together with indirect foraminal decompression, although the correction remained below the implant’s intrinsic lordosis, which is consistent with our findings [8]. Lee DY and Marouby et al. observed SL gains of 7.8° and 4.8° with 12° and 10° implants, respectively [17,18], while Siepe et al. noted significant SL correction but a slight decrease in overall lumbar lordosis [19]. Kapustka et al. demonstrated increases in both foraminal height and L1 to S1 lordosis with anteriorly elevated implants [20]. Similarly, Afathi et al. showed that lordosis gains were mostly limited to the lower lumbar levels, without a major impact on L1 to S1 [21].

The present study found a moderate association between anterior implant height (IAH) and L4 S1 correction, postoperative LDI, and foraminal height. Corrected CR was also associated with FH variation, whereas implant lordosis showed no significant association with postoperative alignment, even though the lowest implant lordosis used was still 10°. These associations should be interpreted as exploratory and do not imply causality, as the heterogeneity of implant designs may represent an additional source of uncontrolled confounding. These findings contrast with the predictive model proposed by Ahlquist et al., which incorporated implant lordosis as a determinant of postoperative SL [6].

In line with our results, Wu et al. reported that implant position along the anteroposterior axis, rather than implant lordosis, influenced segmental and disc height correction [22]. Zavras et al. further stressed the importance of implant positioning, showing that anterior placement in ALIF increased the risk of cage subsidence [23]. Likewise, Issa et al. demonstrated that anterior positioning of LLIF cages was more effective for SL correction, whereas posterior placement favored posterior disc height restoration, again without a consistent correlation between implant lordosis and sagittal correction [24]. Collectively, these findings suggest that implant positioning and height play a more decisive role than implant angulation in sagittal restoration.

Exploratory PI subgroup analysis revealed significant differences in L1-S1 lordosis, but not at the distal levels, suggesting that PI primarily influences proximal lumbar lordosis (L1-L4), as found by Pesenti et al. [25]. However, these subgroup findings should be interpreted with caution, particularly for patients with high PI, as the small sample size in this subgroup limits statistical power and precludes definitive conclusions. An inverse correlation was observed between preoperative PI and postoperative LDI, highlighting the difficulty of restoring harmonious sagittal alignment in extreme PI cases. Specifically, patients with low PI are at risk of overcorrection at L5-S1, whereas those with high PI may remain undercorrected. Based on these observations, implants with lower anterior height may be preferable in low-PI patients to avoid excessive distal lordosis, while higher IAH may be beneficial in high-PI patients to optimize sagittal alignment.

We also observed a postoperative increase in PI, which may reflect sacroiliac joint remodeling, although these differences remain minimal and may be partly attributable to measurement variability. Lee et al. previously described PI increases in patients undergoing long fusions, including the sacrum [26]. Similar mechanisms may explain the occurrence of sacroiliac joint pain following isolated L5-S1 ALIF [27]. This observation deserves further investigation, as it may influence both implant selection and patient counseling.

The present results should be interpreted in light of several limitations. The retrospective, single-center design carries inherent risks of selection bias and limits the generalizability of the findings. Although the sample size is relatively large compared with previous series, it remains modest and may lack sufficient statistical power to detect subtle associations. The analysis was purely radiological and did not include clinical outcomes. The absence of a control group prevents comparisons with other fusion techniques or with posterior instrumentation and therefore precludes technique-specific or causal inferences. Further prospective studies integrating clinical endpoints are required to confirm both the long-term durability of these radiological findings and their clinical relevance. All radiological measurements were performed by a single blinded observer, and the absence of inter- and intra-observer reproducibility testing may have introduced measurement bias. This limitation could be addressed in future studies by involving multiple blinded observers and evaluating reproducibility to enhance the reliability of the measurements.

In addition, follow-up was heterogeneous and relatively short, which precluded the evaluation of long-term durability. Standardized and longer follow-up would be required to more accurately assess the maintenance of sagittal correction and fusion over time. Finally, although EOS imaging offers important advantages, including low radiation exposure and acquisition in a standing position with parallel X-rays that reduce image distortion compared with conventional radiographs, it may still underestimate small segmental variations compared with CT, which remains the gold standard for precise angular measurements. Patients without preoperative or postoperative standing EOS imaging were excluded from the analysis, potentially introducing selection bias, as this subgroup may not be fully representative of the overall population undergoing standalone L5-S1 ALIF.

Several different implant designs were used in this study, including integrated cage-and-plate systems that allow direct continuity with the anterior vertebral wall, as well as zero-profile implants that enable deeper seating to enhance press-fit stability. This heterogeneity represents a limitation because implant characteristics were not fully standardized and may have introduced uncontrolled confounding, thereby reducing the internal validity of the correlation analyses. Implant type was not included as an adjustment variable, and no stratified or multivariable analyses were performed. Consequently, the observed associations between implant parameters and radiological outcomes may be partially influenced by unmeasured implant-related characteristics, limiting internal validity and precluding causal interpretation.

Despite these limitations, this study has important strengths. It focused exclusively on a homogeneous cohort of patients undergoing standalone L5-S1 ALIF for DDD, excluding those with transitional anomalies, spondylolisthesis, or prior instrumentation, thereby reducing potential confounding factors. All patients were evaluated with standardized EOS imaging in the standing position with hands on the shoulders, as well as CT scans, using consistent radiological parameters to ensure reproducibility. These exploratory findings may provide a basis for future research and guide surgical decision-making regarding implant parameter selection in standalone L5-S1 ALIF. However, further prospective studies that incorporate clinical outcomes and standardized implant selection are needed to optimize surgical planning and confirm the clinical relevance of these associations.

## Conclusions

In patients with single-level degenerative disc disease, standalone L5-S1 ALIF was associated with improvements in caudal, segmental, and global lumbar lordosis, as well as with restoration of lumbar lordosis distribution and indirect foraminal decompression. Postoperative L4-S1 lordosis approached PI-based theoretical alignment targets, which were used in this study as a descriptive reference framework rather than as validated surgical endpoints. Exploratory analyses indicated that implant-related parameters were associated with postoperative radiological outcomes. In particular, anterior implant height appeared to be the implant parameter most consistently associated with L4-S1 lordosis correction, lordosis distribution, and FH, whereas implant lordosis itself was not linked to postoperative sagittal alignment. These findings should be interpreted in the context of the retrospective and observational design and do not imply causality. Further prospective studies incorporating clinical outcomes and standardized implant selection are needed to more clearly define the role of implant parameters in optimizing sagittal alignment after standalone L5-S1 ALIF.
